# Ambulatory assessment to predict problem anger in trauma-affected adults: Study protocol

**DOI:** 10.1371/journal.pone.0278926

**Published:** 2022-12-22

**Authors:** Olivia Metcalf, Laura Finlayson-Short, Karen E. Lamb, Sophie Zaloumis, Meaghan L. O’Donnell, Tianchen Qian, Tracey Varker, Sean Cowlishaw, Melissa Brotman, David Forbes

**Affiliations:** 1 Department of Psychiatry, Phoenix Australia, University of Melbourne, Carlton, Victoria, Australia; 2 Centre for Epidemiology and Biostatistics, Melbourne School of Population and Global Health, University of Melbourne, Carlton, Victoria, Australia; 3 Department of Statistics, University of California, Irvine, Irvine, California, United States of America; 4 Neuroscience and Novel Therapeutics Unit, Emotion and Development Branch, National Institute of Mental Health, National Institutes of Health, Bethesda, Maryland, United States of America; Public Library of Science, UNITED KINGDOM

## Abstract

**Background:**

Problem anger is common after experiencing a traumatic event. Current evidence-driven treatment options are limited, and problem anger negatively affects an individual’s capacity to engage with traditional psychological treatments. Smartphone interventions hold significant potential in mental health because of their ability to deliver low-intensity, precision support for individuals at the time and place they need it most. While wearable technology has the capacity to augment smartphone-delivered interventions, there is a dearth of evidence relating to several key areas, including feasibility of compliance in mental health populations; validity of in vivo anger assessment; ability to predict future mood states; and delivery of timely and appropriate interventions.

**Methods:**

This protocol describes a cohort study that leverages 10 days of ambulatory assessment in the form of ecological momentary assessment and a wearable. Approximately 100 adults with problem anger will complete four-hourly in vivo mobile application-delivered micro-surveys on anger intensity, frequency, and verbal and physical aggression, as well as other self-reported mental health and wellbeing measures. Concurrently, a commercial wearable device will continuously record indicators of physiological arousal. The aims are to test the feasibility and acceptability of ambulatory assessment in a trauma-affected population, and determine whether a continuously measured physiological indicator of stress predicts self-reported anger intensity.

**Discussion:**

This study will contribute new data around the ability of physiological indicators to predict mood state in individuals with psychopathology. This will have important implications for the design of smartphone-delivered interventions for trauma-affected individuals, as well as for the digital mental health field more broadly.

## Introduction

More than 70% of adults will experience a traumatic event in their lifetime [1]. Problem anger is one of the most common mental health issues in individuals who have experienced trauma, and is characterised by difficulties recognising early physiological and emotional signs of anger (including altered physiological arousal), swift escalations of affective states, and problems engaging adaptive coping strategies to prevent anger from becoming overwhelming [2]. Problem anger in trauma-affected populations (sometimes referred to as ‘posttraumatic anger’; [3] is often overlooked and rarely addressed relative to other posttraumatic mental health concerns, despite affecting up to 30% of individuals [4, 5]. Problem anger has significant behavioural, social and occupational impacts, including strong links with suicide [5]. Current anger treatments focus on cognitive behavioural therapy (CBT) principles of improving emotional regulation through increased self-awareness and tailored anger control plans that can shift the individual towards greater internal focus and awareness of escalating emotional states, coupled with adaptive coping strategies [6]. Psychotherapy process research has found that of the various CBT components delivered in anger treatments, arousal calming skills consistently predicts improvement [7]. However, such treatments are typically delivered in weekly psychotherapy formats and are associated with high levels of patient drop-out and modest outcomes, in part due to the hostile and irritable nature of individuals with problem anger and the effect of this on the therapeutic alliance [8]. A review on anger management approaches concluded that there is a need for better understanding of characteristics that may impede therapeutic change, in order to refine current treatments, and develop innovative approaches to intervention [9].

Digital mental health interventions offer promise to individuals with problem anger. Recent research has shown that CBT principles can be effectively delivered via smartphone to individuals with problem anger [10], and that longitudinal smartphone-based studies are feasible in individuals with problem anger [11]. Previous research in problem anger has also shown that anger varies frequently on a moment-to-moment basis and, like other affective states, is ecologically influenced. This creates an opportunity for personalised digital interventions such as just-in-time adaptive interventions [12], which have the goal of delivering support “at the moment in the context that the person needs it most and is most likely to be receptive [13]”. In addition to smartphone delivery, modern technology can facilitate multimodal ambulatory assessment and peripheral physiology metrics that can integrate detailed self-report data via ecological momentary assessment with objective, passively collected sensor data through smartphones and wearables. The combined data may provide rich, ecologically valid, contextual, and biopsychosocial information about mental health issues, including temporal information about the dynamics of psychological phenomena [14]. As such, there is the potential for wearable technology to be leveraged to reliably predict and subsequently alter mood states [15]. Given the preliminary findings relating to the potential for digital mental health to address problem anger, coupled with advances in wearable and smartphone technology, the primary aims of this study are to:

1. Investigate the feasibility and acceptability of the use of a commercial wearable to capture physiological data in a trauma-affected population.
2. Determine whether high levels of self-reported anger intensity can be predicted from physiologically measured stress.

Secondary aims from this study will be exploratory in nature. We will examine relationships among common associated features of problem anger such as pain, alcohol use, sleep, and anger rumination, as well as physiological variables collected from the wearable such as heart rate, inter-beat-interval, location, and steps. The findings from this study will provide world-first evidence for the feasibility of ambulatory assessment (i.e., smartphone and wearable) of physiological and emotional aspects of dysregulated mood in trauma-affected individuals, to inform the development of digital mental health tools.

## Materials and methods

### Participants

Adults aged over 18 with problem anger who have experienced a previous traumatic event will be recruited from around Australia (see Table 1). Participants will be recruited through the researcher’s participant database of trauma-exposed individuals, as well as targeted social media adverts.

**Table 1:** Study inclusion and exclusion criteria.

Inclusion	Exclusion
≥18 years	Have a recent history of significant violence
Problem anger (≥12 points on the 5-item Dimensions of Anger Reactions Scale)	Physical health conditions affecting the cardiovascular system
Experienced a previous traumatic event (endorsement of a DSM-5 Criterion A event)	Unwilling to use the wearable for 10 days
Own an internet-connected smartphone	

### Study design

This study is a cohort study using ambulatory assessment (i.e., ecological momentary assessment and a wearable; Garmin Vivofit 4), with data collected at three time points: Time 1: pre-ambulatory self-report surveys; Time 2: ambulatory assessment (for a 10-day period); and Time 3: post-ambulatory self-report surveys and qualitative interviews. Self-report surveys will be completed online using REDCap. Ambulatory assessment data will be collected by participants using a commercial wearable and a customised study app (mEMA-sense), in conjunction with their own personal Android or Apple smartphone. This is an Australia-wide study with data analyses occurring in Melbourne, Australia. As this is a study protocol, no data has been included, conforming to the PLOS data policy.

### Power and sample size

The total feasible sample size is ~100 participants due to the expense of wearables. Specific sample size details for Aim 1 and 2 are described below.

For Aim 1, a target of a minimum of 75% of participants reporting satisfaction with ease of use of the wearable and app, and low frustration is set. A total feasible sample size of 100 participants would ensure that the precision of the two-sided 95% confidence interval of the true underlying proportion of participants who adhere to the intervention is at most +/- 9% when assuming a proportion of 75% using the Wald method to measure the 95% confidence interval.

For Aim 2, a sample size of 100 participants provides a total of 4000 possible outcome observations since each participant has 40 measurement occasions. It is assumed that up to 30% of these observations will be missing [11], resulting in 2800 possible observations (approximately 28 per participant) and that 25% of recorded outcomes will be high intensity anger (≥ 7). The design effect is estimated to be 14, assuming an intraclass correlation of 0.48 from prior data, which leads to an effective sample size of 200 for this study if 100 participants are recruited. Sample size calculations for prediction modelling were conducted according to the three criteria (shrinkage, overfitting and precision; [16]. An effective sample size of 200 results in the ability to detect a minimum Cox-Snell R^2^ of approximately 0.04 assuming one predictor (physiologically recorded stress; 1–100) and ensuring the shrinkage is the recommended 0.9. This sample size satisfies the overfitting criteria for a maximum possible Cox-Snell R^2^ of 0.53, according to estimates of the null model log-likelihood and provides a margin of error of 0.06, marginally higher than the more stringent 0.05 but below the required 0.1.

### Measures

This longitudinal observational study will collect a wide range of self-reported, physiological, and other sensor data. The measures relating to the primary outcomes will be described first, followed by EMA measures, wearable measures, and other self-reported data.

#### Primary outcomes

For Aim 1, acceptability, feasibility, adherence, and usability will be measured using a mixed methods approach. Acceptability and usability will be collected from participants at Time 3, using quantitative self-report measures of ease of use (1; Very Difficult to 5; Very easy), ease of understanding (1; Very Difficult to 5; Very easy), and levels of frustration with the study protocol (1; Very Frustrating to 5; Not at all), as well as qualitative responses to strengths and limitations of the protocol. Feasibility and adherence will be determined using missing data patterns for both wearable and EMA data, and number of support calls required.

For Aim 2, an EMA measure of anger intensity (*Rate the overall intensity of the anger or irritability you feel right now*; 10-point Likert Scale ‘1’ Lowest intensity; ‘10’ Highest intensity), and wearable data related to physiological indictor of stress (1–100) will be used (see sections below for more details).

#### EMA measures

EMA measures of anger intensity, frequency, verbal aggression, physical aggression, pain, sleep, alcohol use, and rumination will be collected (Table 2). EMA measures will be delivered approximately every four hours with an individualised schedule. Participants will be asked to nominate their daily waking time prior to study commencement, and the first daily EMA is randomised to occur within the one hour of the waking window. From there, the second, third and fourth EMA surveys for the day occur at randomised four hourly intervals. EMA measures are delivered to the individual’s smartphone via the mEMA-sense platform.

**Table 2:** Overview of EMA measurements.

Construct	Item wording	Scale type	Prompting time			
			1	2	3	4
Anger-related rumination	*In the past 3 hours I kept thinking about events that had angered me*	4-point Likert Scale	✓	✓	✓	✓
		(‘1’ Almost Never to ‘4’ Almost always)				
Pain	*How much pain are you currently experiencing*?	Visual analogue scale	✓	✓	✓	✓
		[no pain] to [worst pain imaginable]				
Alcohol consumption	*In the past 3 hours*, *how many standard drinks containing beer*, *wine*, *liquor*, *or any other alcohol did you consume*?	8-point Likert Scale	✓	✓	✓	✓
		*(‘0’ No drinks to ‘7’ 7+ drinks)*				
Sleep quality	*During the past night*, *how would you rate your sleep quality overall*?	4-point Likert Scale	✓			
		(‘*0’ Very good; ‘1’Fairly good; ‘2’ Fairly bad; ‘3’ Very bad*)				
Nightmares	*During the past night*, *how distressing were your dreams or nightmares*?	5-point Likert Scale	✓			
		(‘*0’ No dream or not at all distressing; ‘1’ Slightly distressing; ‘2’ Somewhat distressing; ‘3’ Very distressing; ‘4’ Extremely distressing*				
Anger intensity	*Rate the overall intensity of the anger or irritability you feel right now*.	10-point Likert Scale	✓	✓	✓	✓
		*‘1’ Lowest intensity; ‘10’ Highest intensity*				
State anger frequency/duration	*In the past 3 hours*, *how much of the time did you feel angry or irritable*?	5-point Likert Scale	✓	✓	✓	✓
		(‘*0’ Never; ‘1’ Little; ‘2’ Somewhat; ‘3’ Much; ‘4’ A great deal*)				
Anger expression #1	*When you felt angry or irritable over the past 3 hours*, *to what degree did you speak or shout about your anger or irritability*?	5-point Likert Scale	C✓	C✓	C✓	C✓
		(‘*0’ Never; ‘1’ Little; ‘2’ Somewhat; ‘3’ Much; ‘4’ A great deal*)				
Anger expression #2	*When you felt angry or irritable over the past 3 hours*, *to what degree did you do physical things like gesture*, *pound the table*, *slam doors*, *throw things*, *and so forth*?	5-point Likert Scale	C✓	C✓	C✓	C✓
		(‘*0’ Never; ‘1’ Little; ‘2’ Somewhat; ‘3’ Much; ‘4’ A great deal*)				

#### Wearable data

Wearable outcomes will include heart rate, inter-beat interval, stress, daily steps per day, and GPS location at the time of the EMA prompt. The key wearable variable of interest for Aim 2 is stress.

#### Demographic and mental health measures

In addition to EMA and wearable data, self-report measures for pre- and post-ambulatory assessment will be collected. These are presented in Table 3.

**Table 3:** Overview of pre- (Time 1) and post- ambulatory assessment (Time 3) measures.

Time point(s)	Construct	Measure
Time 1	Demographic information	Gender, age, relationship, employment, treatment information
Time 1; Time 3	Problem anger	Dimensions of Anger Reactions Scale-5 (DAR-5; a cut-off of ≥12).
Time 1; Time 3	State and trait anger	State-Trait Anger Expression Inventory 2 (STAXI-2)
Time 1; Time 3	Pain	Visual analogue scale (VAS)
Time 1; Time 3	Posttraumatic stress disorder symptoms	Posttraumatic Stress Disorder Checklist (PCL-5)
Time 1; Time 3	Emotional self-awareness	Emotional Self Awareness Scale (ESAS)
Time 1; Time 3	Anger rumination	Anger Rumination Scale (ARS)
Time 1	Trauma exposure	Life Events Checklist for DSM-5 (LEC-5)
Time 1	Sleep disturbances	Insomnia Severity Index (ISI)
Time 1	Alcohol use	Alcohol Use Disorders Identification Test-Concise (AUDIT-C)

#### Data collection and management

Data will be collected through three channels, including REDCap for pre- and post-study surveys, wearable sensors, and smartphones. Wearable data will be transferred via Bluetooth to the individual’s smartphone, and smartphone data will then be pushed to the cloud-based platform ilumivu (ilumivu, Asheville, NC). Data in this platform is encrypted and anonymised and will be downloaded into the local database once data collection is finished. Access to both cloud and local databases is password-protected so entry is permitted to study investigators only.

#### Safety considerations

During the EMA assessment period, participants will be prompted on a regular basis to consider their anger and aggression. Previous research in this population indicates that regular prompting does not increase distress or anger [11]. To mitigate risk of working with individuals at significant risk of violence to others, individuals are screened out if they have recently used significant violence (i.e., resulting in injury to others, used a weapon, and/or used choking in the past six months). In addition, the study employs a clinical psychologist and psychiatrist who oversaw the development of a risk management plan for participants to monitor and manage risk of harm to self and others.

#### Statistical analyses

For Aim 1, descriptive analyses of quantitative measures of acceptability, feasibility, adherence, and usability will be conducted and mean, range and median responses to questions reported. Qualitative data collected from interviews with participants will be synthesised.

For Aim 2, continuously measured physiological stress (Garmin formula [17]; score 1–100) captured by wearable sensors will be examined as a predictor of self-reported anger intensity (1–10). Values recorded in the 10-minute interval prior to the EMA micro-survey will be extracted for use in the prediction models.

Descriptive analyses will be performed on the data to determine missingness, completion rates, outliers and erroneous values, and a proxy for wear time of the wearable sensor, e.g. time between first and last values each day [18]. The following analysis methods that can handle predictors that have been continuously measured prior to each longitudinal outcome measurement will be considered: i) two-stage approaches where appropriate features (maximum, variance, etc.) of the continuously measured predictors are included in a generalised linear mixed model [18–20]; ii) functional data analysis (FDA) approaches, where the continuously measured predictors are treated as functions when building prediction models [21]. FDA can be implemented in either regression modelling frameworks [22] or machine learning frameworks, such as functional partial least squares [23] and random forests [24]. The predictive performance of each model will be assessed using the balanced error rate (percentage of misclassified momentary anger episodes) calculated using 5-fold cross-validation. Variables importance will also be assessed using metrics suitable for the modelling approach [25].

Additional models, including Dynamic Structural Equation Models (DESM) and Time Varying Effects Models (TVEM), will be built to investigate bi-directional relationships between anger and variables of pain, alcohol, sleep quality, and rumination. Data cleaning and statistical analyses will be performed in SPSS (v23, IBM) and R (R. RStudio, Boston, MA, USA; [26].

#### Ethics

This research was approved by the University of Melbourne Human Research Ethics Committee (HREC); approval number 2022-22157-28033-5. Written informed consent will be obtained electronically.

#### Status and timeline of the study

Recruitment of participants began on the 1^st^ of August 2022 and will continue for approximately one year. At the time of writing, approximately 15% of the desired sample size has completed the study.

## Discussion

This protocol describes a study designed to investigate the acceptability, feasibility, adherence, and usability of wearable devices to understand anger in people experiencing problem anger. This study will also investigate whether wearable collected physiological data can predict anger intensity. This study will offer key data components to deconstruct the complex interactions between anger, and individual and contextual factors, in trauma-affected individuals. Relevant data collected includes daily experiences of anger and aggression, indicators of wellbeing such as pain, subjective sleep quality, alcohol use, and rumination, combined with physiological markers, including cardiovascular and physical activity as well as objective sleep metrics and GPS location data. The primary questions this study will answer are whether ambulatory data (i.e., wearable and smartphone) collection is feasible and acceptable in trauma-affected populations and whether physiological data collected from a wearable can reliably predict anger or aggression. Answers to these questions will inform the development of digital mental health assessment and intervention tools, such as just-in-time adaptive interventions, to trauma-affected populations. More broadly, the potential for physiological data collected via a commercial wearable to predict mood state holds significant value across a range of psychiatric conditions that are characterised in terms of dysregulated mood states. This is particularly pertinent in the treatment of problem anger in that arousal-calming skills are related to symptom improvement [7], and physiological data holds rich arousal related information.
